# An optimization method for parameter extraction of metals using modified Debye model

**DOI:** 10.1186/2193-1801-2-426

**Published:** 2013-08-30

**Authors:** Rakibul Hasan Sagor, Md Ghulam Saber, Md Thesun Al-Amin, Asif Al Noor

**Affiliations:** Department of Electrical and Electronic Engineering, Islamic University of Technology, Board Bazar, Gazipur, 1704 Bangladesh

**Keywords:** Finite-difference time-domain, Modified Debye model, Metal optics, Near-IR photonics

## Abstract

The Modified Debye Model (MDM) parameters for five metals are presented. A nonlinear optimization algorithm has been developed in order to extract the parameters for the metals. The extracted parameters have been used to determine the complex relative permittivity of the metals in optical and near-IR region of electromagnetic spectrum. The obtained results have been compared with the experimental values and an excellent agreement has been found.

## Introduction

The finite-difference time-domain (FDTD) method, proposed by Yee (1966), is one of the widely used algorithms in computational electromagnetics. The time dependent form of Maxwell’s equations can be successfully integrated in this method. The major advantage of the time-domain method is that the solutions can cover a wide frequency range with a single simulation run. This reduces the computation time and memory requirement significantly.

Original formulations of Yee described the isotropic materials with static permittivity only. However, in order to simulate real dispersive materials using FDTD method, we need to incorporate the formulations of frequency dependent properties of materials in the simulation model.

Choice of ohmic contacts dramatically influences the performance of optoelectronic devices. Metallic mirrors serve as ohmic contacts as well as reflectivity enhancer in lasers and LEDs (Baba et al. 1996; Chang-Hasnain et al. 1991; Hunt et al. 1993; Katz 1995; Luo and Zory 1990; Smith et al. 1995). It has been observed that wetting metals like Palladium and Titanium provide reliable adhesion and better ohmic contacts (Yang et al. 1990). The shortcomings of noble metals like gold can be overcome by employing a thin layer of Titanium on the surface. Besides, metals are indispensable for producing surface plasmon polariton (SPP). Thin Nickel films have drawn a lot of attention of the researchers for producing long-range SPP (Zervas 1990; Yates et al. 2006; Hickernell and Sarid 1987). Therefore, modeling parameters of these metals should be available in order to simulate and investigate new techniques for optoelectronic devices. Oftentimes researchers consider perfect electric conductors in their simulation model due to the lack of appropriate modeling parameters of metals (Shi and Hesselink 2004; Shi et al. 2002).

The parameters of several metals have been reported to our knowledge. Jin *et al.* have determined the MDM (Kunz and Luebbers 1993) parameters for gold which are applicable in the wavelength range of 550–950 nm (Jin and Xu 2006). Krug *et al.* have reported the gold parameters that are applicable in the wavelength range of 700-1000 nm (Krug et al. 2002). W.H.P. Pernice *et al.* (Pernice et al. 2007) have extracted the parameters for Nickel using Lorentz-Drude model. A.D. Rakic *et al.* (Rakic et al. 1998) have reported the parameters for Nickel, Palladium, Titanium and 8 other metals using Lorentz-Drude and Brendel-Bormann Model. M.A. Ordal *et al.* (Ordal et al. 1985) have extracted the parameters for fourteen metals in the infrared and far-infrared range. However, extraction of parameters using modified Debye model (MDM) (Kunz and Luebbers 1993) for these metals have not been reported to our knowledge.

Herein, we focus on real metals which exhibit dispersive properties at high frequency. In order to integrate the frequency dependent dispersive properties of materials in the FDTD algorithm, the constitutive parameters should be denoted as constants. We use the modified Debye model (MDM) (Kunz and Luebbers 1993) to describe the frequency dependent dispersive behavior of the real metals. However, the parameters need to be extracted in order to include them in the FDTD formulations. We develop a nonlinear algorithm to extract the parameters for Nickel, Hexagonal Cobalt, Palladium, Iridium and Titanium. The obtained parameters have been used to determine the complex relative permittivity of the metals which have been compared with the experimental values (Palik 1998). From our comparison we find that the RMS deviations for the extracted parameters are as little as 1.0270, 1.1519, 0.2489, 1.6216 and 1.1239 respectively. The advantage of our extracted parameters is that it requires less computation time and exhibits less RMS deviations from the parameters reported by other researchers.

## Material model and optimization method

### Material model

The complex relative permittivity function of the modified Debye model (Kunz and Luebbers 1993) is described by the following equation,1

where, *ϵ*_*∞*_ is the infinite frequency relative permittivity, *ϵ*_*s*_ is the zero frequency relative permittivity, *ω* is the angular frequency, *τ* is the relaxation time and *σ* is the conductivity.

If the model is represented in terms of its real and imaginary parts, then,2

where, the real part of the complex relative permittivity is,  and the imaginary part of the complex relative permittivity is, .

From (1), we can see that the modified Debye model can be described by four parameters which are *ϵ*_*∞*_, *ϵ*_*s*_, *τ* and *σ*. However, a relationship can be derived among these parameters by comparing (1) with the Drude model equation as,3

Now we actually have three parameters that need to be extracted and the other can be obtained from (3).

### Optimization method

The equation describing the modified Debye model is nonlinear in nature which is why it is difficult to develop an optimization algorithm that can perfectly extract the modeling parameters. However, we have developed a minimization algorithm that utilizes the least-squares technique.

The algorithm that we have used is as follows. First we obtain the experimental values from the book of Palik (Palik 1998) and use them to obtain the complex relative permittivity for each material. Then the program varies the three parameters that need to be extracted and try different combinations to obtain the complex relative permittivity. The square of the complex relative permittivity obtained using the extracted parameters are subtracted from the square of the complex relative permittivity obtained using experimental values. The squared differential term is then compared with a predetermined tolerance value which is near to zero and the iteration goes on until the preset value is reached. The variation in the modeling parameters is random; however, boundary limits have been set so that the extracted parameters meet the requirement of the FDTD method. Varying the modeling parameters in a random fashion is the most challenging part of the algorithm. If a linear method was used to vary the parameters, the computation time would have been much higher. Since the variation is random, it takes less time to find a combination of values that produces the least squared difference.

The boundary conditions that need to be maintained for the MDM parameters to be integrated in the FDTD algorithm are *ϵ*_*∞*_ > 1, *ϵ*_*s*_ < *ϵ*_*∞*_ and *σ* ≥ *ϵ*_*o*_(*ϵ*_*∞*_ − *ϵ*_*s*_)/*τ*.

**Figure 1:**
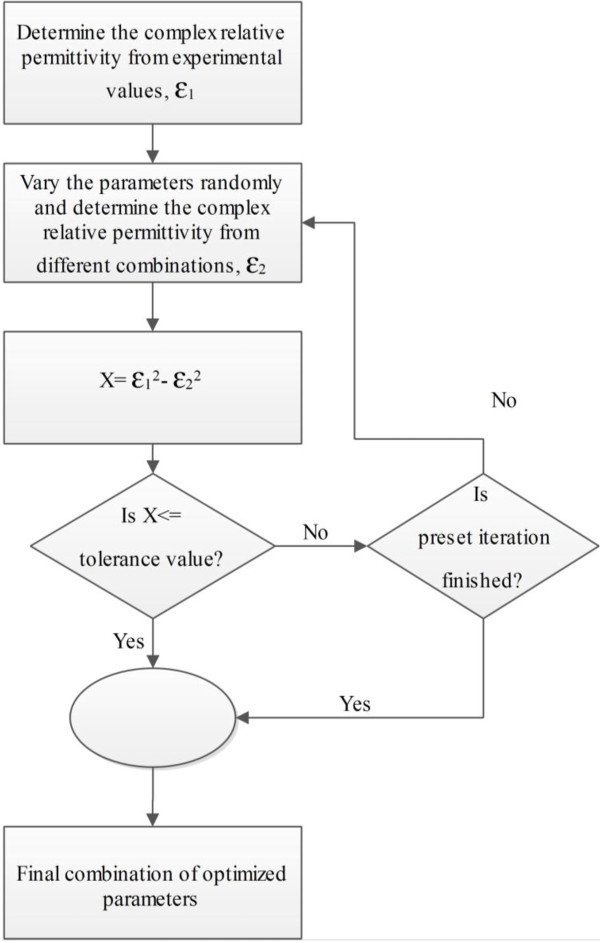
**Flow chart of the optimization algorithm used for material modeling parameter extraction.**

The flow chart of the optimization algorithm is given in Figure 1. The algorithm has been developed solely for single-pole modeling parameter extraction. The accuracy of the obtained results reduces significantly if the modeling parameters for more complex curves are extracted using this optimization algorithm.

## Results and discussion

The extracted parameters for the five metals using our developed algorithm are presented in Table 1. From the table it can be observed that a maximum RMS deviation of 1.62 occurs for Iridium which indicates the robustness and accuracy of our optimization algorithm.

**Table 1:** **Extracted modified Debye model parameters for metals**

Parameters	Nickel	Hexagonal cobalt	Palladium	Iridium	Titanium
*ϵ*_*∞*_	2.2986	1.0001	1.0010	1.0001	7.5900
*ϵ*_*s*_	−22.52	−25.65	−22.00	−40.92	−11.99
*σ* (S/m)	6.74 x 10^5^	7.86 x 10^5^	7.53 x 10^5^	1.01 x 10^6^	6.21 x 10^5^
*τ*_(sec)_	3.26 x 10^-16^	3.00 x 10^-16^	2.70 x 10^-16^	3.70 x 10^-16^	2.79 x 10^-16^
Range of wavelength (μm)	0.6-1.1	0.35-1.0	0.3-0.7	0.6-1.1	0.4-0.7
RMS deviation	1.027	1.1510	0.2489	1.6216	1.1239

**Figure 2:**
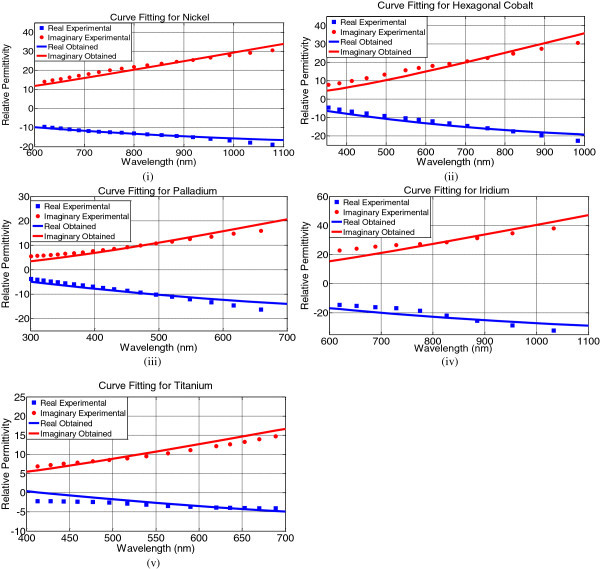
**Comparison between the real and the imaginary parts of the complex relative permittivity obtained using both extracted parameters and experimental values for (i) Nickel (ii) Hexagonal Cobalt (iii) Palladium (iv) Iridium (v) Titanium.**

The complex relative permittivity for each metal has been determined using both extracted parameters and experimental values. Then the real and imaginary parts have been separated from the complex relative permittivity and plotted which is presented in Figure 2(i,v). The red color indicates the imaginary part while the blue color indicates the real part of the complex relative permittivity. The solid lines denote the extracted parameters and the dotted lines denote the experimental values. From the figure it is clearly visible that the real and imaginary parts of the complex relative permittivity obtained using extracted parameters agree very well with the real and imaginary parts of the complex relative permittivity obtained from the experimental values (Palik 1998).

The parameters obtained by Rakic *et al.*(Rakic et al. 1998) for Nickel exhibits an RMS deviation of 4.38 in the wavelength range of 0.6 to 1.1 μm and the parameters obtained by Pernice *et al.* (Pernice et al. 2007) for Nickel show an RMS deviation of as large as 24.11 whereas our obtained parameters show an RMS deviation of only 1.02. For Palladium, the RMS deviation for the parameters reported by Rakic *et al.*(Rakic et al. 1998) is 3.1251 while it is only 0.25 for our case within the wavelength range of 0.3 to 0.7 μm.

The optimization method we have developed adopts random variation of the modeling parameters which reduces the computation time significantly while maintaining an excellent degree of accuracy for the single-pole material models. This is evident from the RMS deviation comparison of our extracted parameters with the results reported by other researchers.

We have utilized single-pole Debye model to fit our curves for the materials while Rakic *et al*. (Rakic et al. 1998) have used a six-pole Lorentz-Drude model and Pernice *et al*. (Pernice et al. 2007) have used a four-pole model for the fitting purpose of the frequency dependent complex permittivity curve of metals. The more is the number of poles, the higher is the computation time required. Our single-pole model requires significantly less computation time in comparison to multiple-pole models. We have simulated both single-pole and six-pole models for Nickel in an intel Core v i5 processor based computer and found that the computation time is reduced by ~15% using our extracted parameters. The difference in the computation time would be even more noticeable for longer distances of propagation. Therefore, it is evident that our results are better than theirs in terms of both accuracy and computation time.

## Conclusion

We report the modified Debye model parameters for five metals obtained using a nonlinear optimization algorithm which are valid for a wide frequency range. The extracted parameters are expected to be useful for the integration of material properties in the simulation model and obtain more accurate results. The accuracy of the parameters has been verified by comparing them with the experimental values.
